# Predictive Capacity of Different Indicators of Adiposity for Metabolic Syndrome in Adults in the City of Trujillo, Peru

**DOI:** 10.3390/medicina61030419

**Published:** 2025-02-27

**Authors:** Jorge Luis Díaz-Ortega, Joao Caballero-Vidal, Irma Luz Yupari-Azabache, Juan M. Alva Sevilla, Nelson Enrique Conde-Parada

**Affiliations:** 1 Escuela Profesional de Nutrición, Universidad César Vallejo, Trujillo 13001, Peru; alonzo@ucvvirtual.edu.pe; 2 Institutos y Centros de Investigación, Universidad César Vallejo, Trujillo 13001, Peru; iyupari@ucv.edu.pe; 3 Escuela Profesional de Medicina, Universidad César Vallejo, Trujillo 13001, Peru; jalvas@ucv.edu.pe; 4 Programa de Nutrición y Dietética, Universidad Mariana, San Juan de Pasto 520001, Colombia; nconde@umariana.edu.co

**Keywords:** dyslipidaemias, metabolic syndrome, obesity, triglycerides, lipoprotein

## Abstract

*Background and Objectives*: Various adiposity indicators have been used to predict metabolic syndrome (MetS). The aim of the present study was to evaluate the predictive ability of known adiposity indicators, such as abdominal girth, girth/height and fat percentage, as well as less commonly used indicators, such as the conicity index (CI), body roundness index (BRI), visceral adiposity index (VAI) and body shape index (ABSI), to predict MetS. *Materials and Methods*: A total of 261 participants, including family members and graduates of a flagship school in the city of Trujillo, Peru, participated. Metabolic syndrome was assessed according to the harmonised ATP III criteria. ROC curves were analysed for each of the adiposity indicators using SPSS 26.0 statistical software. *Results*: The prevalence of MetS was found to be 43.4%, with a higher proportion in men (25.8%). The area under the curve (AUC) for the prediction of MetS exceeded a value of 0.8 for VAI, abdominal circumference, circumference/height and relative fat mass in both men and women, with VAI showing the highest values of 0.858 and 0.875 in women and men, with cut-off points for MetS of 2.57 and 1.73, respectively. *Conclusions*: VAI can be used in the diagnosis of metabolic syndrome during lipid profile and anthropometric assessment.

## 1. Introduction

Metabolic syndrome (MetS) is a complex pathology that includes five risk factors: abdominal obesity; diabetes; hypertension; and, in the case of dyslipidaemias, hypertriglyceridemia and low levels of low-density lipoprotein (HDL-c) [1].

The prevalence of MetS varies by definition, ranging from 12.5% (ATP III) to 31.4% (JIS); increases with age and year of study; and is higher in urban areas and in women. Prevalence also tends to be higher in higher income countries, being most significant in the Eastern Mediterranean Region (34.6%) and the Americas (33.4%), with the lowest prevalence of MetS occurring in Africa (23.1%). For Europe, it is 31.5% [2].

In Peru, several studies have found prevalences of MetS ranging from 30% to 48% [3,4,5]. Obesity has also been found to be the most prevalent component of MetS [6].

The adipose tissue under the skin is referred to as subcutaneous adipose tissue (SAT), while that overlying internal organs is referred to as visceral adipose tissue (VAT). There are considerable anatomical differences in the distribution of these two adipose tissues in the body. VAT is mainly present in the mesentery and omentum and drains directly to the liver via portal circulation [7]. Another important aspect of VAT is that there is an increased uptake of glucocorticoids and androgens, thereby stimulating lipolysis, ultimately establishing insulin resistance in that tissue and in other organs close to the VAT [8,9].

The body mass index (BMI) is the classic index for defining and classifying obesity; however, it does not measure visceral adiposity [10]. Waist circumference (WC) is the most commonly used anthropometric measure to identify visceral adiposity [11,12]. However, it has been shown that this is not a good indicator to differentiate visceral from subcutaneous fat [10,11].

Relative Fat Mass (RFM), based on the height/waist circumference ratio, is a more accurate method than BMI for estimating the percentage of total body fat among adult individuals [13]. It has been shown that RFM had better predictability than BMI for abnormal low-density lipoprotein (LDL), high-density lipoprotein (HDL) and triglyceride levels, as well as for MetS [14].

The visceral adiposity index (VAI) is a gender-based mathematical equation using biochemical indices including high-density lipoprotein cholesterol (HDL-C), triglycerides (TG) and anthropometric indices (in this case, BMI and WC). This index has been shown to adjust well to visceral adiposity measured by imaging techniques and has been considered a simple surrogate marker of adipose tissue dysfunction and an indirect predictor of cardiometabolic risk [15].

The body roundness index (BRI) is a more accurate mathematical estimation ratio for total body fat and visceral adipose tissue mass by combining height and WC. The body shape index (ABSI), based on BMI, WC and height measurements, is associated with increased abdominal adipose tissue mass and has been reported to be a major risk factor for premature death [16].

The conicity index (CI), is so named because people who accumulate fat around the abdominal region develop the shape of a double cone with their bases united. This indicator relates measures of weight, height and WC and is considered to be the best parameter for identifying fat accumulation. However, like the other adiposity indicators, the CI is not popular among health professionals—even more so due to the complexity of its mathematical calculation [17].

The aforementioned indicators have been used in various investigations in comparison with the best-known anthropometric measurements to assess their predictive capacity for MetS.

Raya Cano et al. [18] observed that the variables with the best ability to discriminate the presence of MetS were the body roundness index (BRI), waist-to-height ratio (WHtR) and WC, compared to other classical anthropometric indicators, such as BMI, waist-to-hip ratio (WHR) and fat percentage, and to other new anthropometric indicators, such as VAI, ABSI and CI. Both WHtR and BRI had higher specificity and sensitivity against SM. A similar result was found in a study by Xu et al. [19], although they did not assess VAI.

However, Baveicy et al. [16] found VAI to be a better predictor of MetS than BRI in adults, which was not observed with ABSI. Likewise, in the case of BRI, it was only a good predictor in men but not in women.

At the national level and in the region of La Libertad—specifically, in the city of Trujillo—no studies have considered this type of indicator, so the determination of its cut-off points will be very useful for the characterization of the study population and for the early detection of MetS.

The works discussed above used the AUC, specificity and sensitivity to compare adiposity indicators; however, a better analysis of predictive ability is needed, especially in those with similar area under the curve (AUC) values, using other probability estimates, such as the positive predictive value (PPV), negative predictive value (NPV) and Youden index.

The aim of this study was to determine the predictive capacity of different anthropometric indicators of adiposity for MetS in adults in the city of Trujillo according to gender, using ROC curve analysis and predictive probability estimators.

## 2. Materials and Methods

### 2.1. Research Design

The present study is of cross-sectional and correlational design.

### 2.2. Population, Sample and Sampling

The population consisted of all the graduates belonging to an emblematic educational institution in the city of Trujillo, as well as their families. The participants are located in different parts of the city, making the sample s representative of the population; however, as it is unknown, the formula was used to calculate the sample from an infinite population with an error of 6% and 95% reliability, resulting in 267 participants [20].

**Figure 1:**
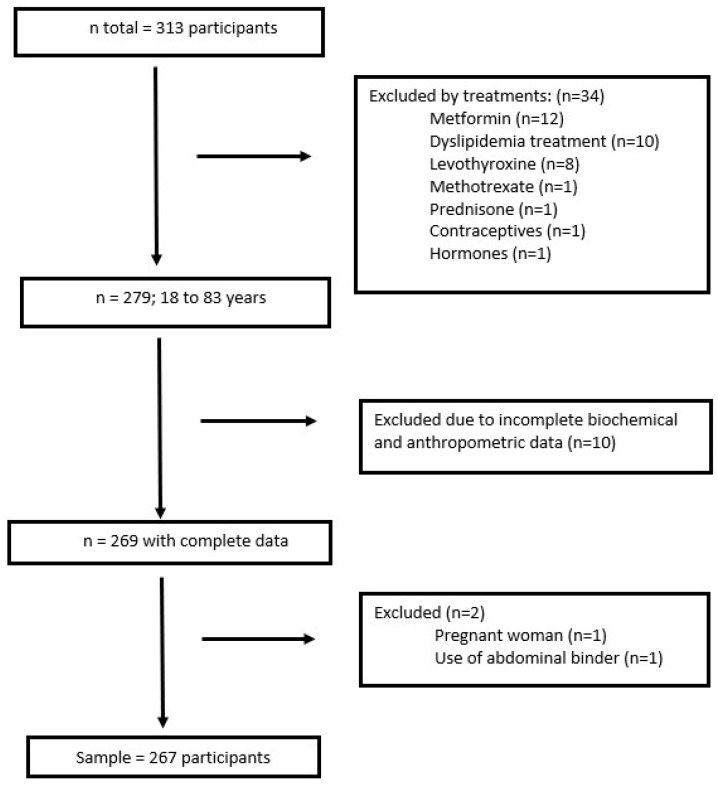
Flow chart of inclusion and exclusion of subjects.

People over 18 years of age, healthy or with comorbidities, were included, with an initial number of 313 recruited. Those treated with drugs for dyslipidaemia (10), metformin (12), levothyroxine (8), corticoids (one case with prednisone), methotrexate (1), menopausal hormones (1) and contraceptives (1) were excluded, as all these drugs affect the lipid profile. Also excluded were those who had incomplete biochemical and anthropometric data (10), were pregnant (1), or had some physical limitation or other aspect that made it impossible to evaluate them (1). In the end, 267 participants remained for data analysis, as summarized in Figure 1.

A non-probability convenience sampling method was used. The president of the alumni association of the educational institution used the alumni register to communicate by email, text message and/or Facebook. Participants who showed interest were registered by the researchers, as were their family members, and prior communication was established to orient them to the purpose of the research and the biochemical analyses and to reserve the date of their assessment.

### 2.3. Assessment of Anthropometric Parameters

The assessment of weight, height, BMI and WC was carried out following the guidelines of the Technical Guide for Anthropometric Nutritional Assessment in Adults [21] and Older Adults [22]. Initial weight measurement was performed using a scale (SECA 813, Hamburg, Germany) while height was recorded with a stadiometer (Seca 213, Hamburg, Germany).

In addition, a caliper skinfold (Harpenden, Burgess Hill, England) was used to assess total body fat. Three accredited nutritionists participated in these anthropometric measurements. To calculate body density, the Durnin and Womersley formula was applied, and to determine body fat percentage, the Siri formula, which takes into account body density, was used [23]. Anthropometric data were recorded on the data collection form (Supplementary Materials).

### 2.4. Assessment of Adiposity Indicators

To assess adiposity indicators, such as BMI, WHtR, CI, ABSI, BRI and VAI, the following calculation equations were applied [18]:

$$BMI=\frac{Weight\;(kg)}{{Height\;(m)}^{2}}$$

WHtR=WC (cm)/Height (cm)

$$CI=\frac{Waist\;Circumference\;(m)}{0.109\times \sqrt{\frac{Body\;Weight\;(Kg)}{Height\;(m)}}}$$

$$ABSI=\frac{Waist\;Circumference}{{BMI}^{2/3}\times {Height}^{1/2}}$$

$$BRI=364.2-365.5\times \sqrt{1-\frac{{\left(\frac{Waist\;Circumference}{2\pi }\right)}^{2}}{{\left(0.5\times Talla\right)}^{2}}}$$

For the ABSI and BRI formulae, WC and height are indicated metres (m).

$$VAI\;men=\frac{Waist\;Circumference}{39.68+(1.88\;BMI)}\times \frac{TG}{1.03}\times \frac{1.31}{HDL}$$

$$VAI\;women=\frac{Waist\;Circumference}{36.58+(1.89\;BMI)}\times \frac{TG}{0.81}\times \frac{1.52}{HDL}$$

For the VAI formula, WC is in centimetres (cm), and triglyceride and HDL concentrations are in mmol/L.

Relative fat mass was considered as described by Kobo et al. [14]:

$$Relative\;fat\;mass\;\left(men\right)=64-(20\frac{Height}{Waist\;Circumference})$$

$$Relative\;fat\;mass\;\left(women\right)=76-(20\frac{Height}{Waist\;Circumference})$$

where height and WC are both in m.

Anthropometric assessments were performed immediately after the biochemical tests for the identification of metabolic syndrome at the Nutritional Assessment Laboratory of the School of Nutrition of the Universidad César Vallejo. All data for adiposity indicators were recorded on the data collection form (Supplementary Materials).

### 2.5. Identification of Metabolic Syndrome

Participants fasted for 10 h at the Research Laboratory of the School of Health Sciences of the César Vallejo University from 8 a.m. on Fridays and Saturdays during the period from September to December 2023. The patient was instructed to rest for approximately 5 to 10 min. To obtain the sample, the fingertips of the index or middle finger were pre-cleaned with 96° alcohol. A puncture was then made with a retractable lancet, and light pressure was applied to collect 35 μL of blood using a capillary tube. This sample was placed upright in the central hole of the test strip, which had previously been inserted into the cholesterol monitoring kit (Mission^®^, Acon Laboratories, San Diego, CA, USA). The results of the lipid profile were expected and included the following measurements: total cholesterol, LDL (low-density lipoprotein), HDL (high-density lipoprotein) and triglycerides. These values were recorded on the data collection sheet.

Normal values for these measurements were as follows: total cholesterol: ≤200 mg/dL; triglycerides: ≤150 mg/dL; LDL: ≤100 mg/dL; HDL for men: >40 mg/dL; HDL for women: >50 mg/dL [24]. Values that were not within the established ranges were considered cardiovascular risk.

Blood from the same finger that was used to measure cholesterol was used to assess glycaemia.

The sample was placed on the test strip previously inserted into a glucometer (Accu-Chek^®^ Performa Nano, Roche, Mannheim, Germany). Fasting glucose was considered normal when values were less than 100 mg/dL.

For blood pressure, a Ri-champion N automated blood pressure monitor was used.

For nutritional status, abdominal circumference was assessed with a metallic tape measure (Lufkin W606PM, Queretaro, Mexico).

For the diagnosis of MetS in the participants, the harmonised ATPIII criteria [25] were used, in which the presence of three of the following risk factors is sufficient to indicate such a pathology: abdominal obesity with WC ≥ 94 cm in men and ≥88 cm in women and, according to the consensus of the Latin American Diabetes Association (ALAD) and according to the Latin American population, high fasting glycaemia (>100 mg/dL), hypertriglyceridaemia (>150 mg/dL) with or without treatment for dyslipidaemia, low HDL concentration with or without treatment or high blood pressure (SBP ≥ 130 mmHg and/or DBP ≥ 85 mmHg) with specific treatment [25,26]. Data were recorded on the data collection form (Supplementary Materials).

### 2.6. Statistical Analysis

The SPSS version 26.0 statistical programme was used to analyse the research data [27]. For descriptive statistics, means and their respective standard deviations, medians and interquartile ranges were calculated for each of the adiposity indicators to avoid the influence of extreme values that alter the behaviour of the analysed baseline characteristics.

Continuous data were tested for normal distribution using the Kolmogorov–Smirnov test before inferential analysis [28]. The normality test showed that most variables did not behave normally. Therefore, the Mann–Whitney U test was used to test for differences between the values of quantitative variables in people with and without MetS.

ROC (receiver operating characteristic curve) analysis was performed to determine the cut-off point of the continuous data for each adiposity indicator, as well as the highest sensitivity and specificity for MetS, using the Youden index [29].

### 2.7. Ethical Considerations

The present research was previously approved by the Ethics Committee of the Professional School of Nutrition of the Universidad César Vallejo with report PI-CEI-NUT-2023-004. The ethical principles established in the Declaration of Helsinki [30] were applied, as well as those contemplated in the code of ethics of the Universidad César Vallejo. These principles include beneficence, non-maleficence, autonomy and justice.

To ensure the voluntary participation of individuals, informed consent was requested. Each participant received detailed information about the objectives of the study and the basic protocols of the analyses to be performed. In addition, knowledge of the researcher’s institutional affiliations was provided.

This process ensured that participants fully understood the information and could freely decide whether to accept or decline to participate in the research, especially if they felt any discomfort.

## 3. Results

### Baseline Biochemical, Physiological and Anthropometric Characteristics of Male Participants

Table 1 shows that there are significant differences (*p* < 0.05) between the baseline characteristics analysed for male participants according to the presence of MetS, with higher mean and median values found in those with a diagnosis of MetS, except for LDL, PCB and height, where similar values were found (*p* > 0.05). In both groups—with and without MetS—the mean and median HDL values are below 40 mg/dL but significantly lower in the group with MetS. LDL concentration was not significantly different between those with and without MetS, and both groups had mean and median values above the threshold of 100 mg/dL and close to 120 mg/dL. For skinfolds, men with MetS had higher median values than those without MetS—1.1, 1.2, 1.3 and 1.5 times more in the bicipital, subscapular, tricipital and suprailiac skinfolds, respectively.

Table 2 shows that in the behaviour of the baseline characteristics analysed in the female participants, there are also significant differences (*p* < 0.05) according to the presence of MetS, with higher averages and medians in those with a diagnosis of MetS, except for LDL and height, which do not differ significantly (*p* > 0.05). In both groups—with and without MetS—HDL has lower mean and median values <50 mg/mL, although significantly lower in the group with MetS. LDL is also elevated in both groups, with means and medians around 130 mg/dL and 144 mg/dL for those without and with MetS, respectively. With respect to skinfolds, it was higher in the MetS group compared to those without MetS; 1.3 times higher in the bicipital skinfold, suprailiac and subscapular skinfolds and 1.2 times higher in the tricipital skinfold.

Table 3 shows that, among the analysed variables, gender is associated with the presence of MetS, with a higher percentage (25.8%) of males having this diagnosis. In terms of age, there is no difference between the proportion of those under 50 years of age and those aged 50 and over with a diagnosis of MetS.

In Table 4, the adiposity indicators with the best area under the curve (AUC) values and higher than 0.8 in the male and female genders correspond to VAI, WC, BRI, RFM and WHtR. VAI had the highest AUC value in both males and females, with the cut-off point for MetS in males being 1.73 and that in females being 2.57. Indicators with AUC values between 0.7 and 0.8 are found in CI and fat percentage in both sexes, showing adequate predictive ability for MetS. BMI is also a good predictor of MetS but does not exceed the AUC value of 0.8 in men, and the BMI cut-off point for MetS in men is close to 28 kg/m^2^, while that in women is approximately 27 kg/m^2^. The ABSI was the least predictive of MetS. Regarding the sensitivity of the test for the positive diagnosis of MetS in men, the ABSI, fat percentage and WHtR (in that order) stand out the least, with low values, while in women, only the ABSI stands out. In terms of specificity in relation to the probability of a negative diagnosis in both men and women, all indicators have acceptable values, although to different degrees.

Finally, VAI is the best indicator, with a high PPV and NPV for MetS and a probability of success of more than 80% in both cases for men, while for women, it has the best PPV compared to the other indicators, with a probability of almost 64% and a very good NPV of almost 90%. This is reflected in the overall performance of the indicator in terms of its discriminative capacity for the diagnosis of MetS with the Youden index, in which VAI in men is the most important among the indicators, with 0.64, while in the case of women, the VAI is 0.6. However, in women, BRI, relative fat mass and WHtR demonstrate a similar discriminative capacity for MetS, although surpassed by WC (0.65); but in all of them, their PPV is below 60%.

## 4. Discussion

It has been determined in other studies, such as those by Ramezankhani et al. [31], Zou [32] and Datta Banik [33], that, in men with MetS, risk parameters are higher than in those without MetS; thus, median glycaemia values can be above 100 mg/dL, triglycerides between 192 mg/dL and 206 mg/dL and HDL between 33 and 43 mg/dL. Systolic blood pressure ranged from 128 to 138 mmHg, while diastolic blood pressure ranged from 80 to 84 mmHg and WC ranged from 100 to 103 cm. The findings of the present work differ from these studies in terms of lower median values of HDL, as well as triglycerides, although exceeding the threshold of 150 mg/dL, and lower values for systolic and diastolic blood pressure but within the acceptable range.

In the case of women with MetS in the studies discussed above [31,32,33], median glycaemia values were also slightly higher than 100 mg/dL, triglycerides had medians between 160 mg/dL and 195 mg/dL, median HDL ranged between 40 and 46 mg/dL; systolic blood pressure was in the range of 126 to 133 mmHg and diastolic blood pressure ranged from 72 to 82 mmHg, while the median WC was 90 to 101 cm. Differences were, again, found with respect to HDL, with a lower value in this range, and with respect to systolic/diastolic blood pressure, for the median was lower, at 120/80 and, therefore, mostly in the normal range. In both men and women, hypertriglyceridaemia and low values of the protective HDL are very important aspects that lead to the development of insulin resistance [34] and, thus, to its presence in MetS [35].

The prevalence of MetS observed in Table 3 is slightly higher than that found by Tejada et al. [4] in a previous study in the city of Trujillo in 2020 and higher than that found elsewhere, including in terms of gender; for example, Sigit et al. [36] found prevalences of 39 and 29% in Indonesia and the Netherlands, respectively, with a higher prevalence in women in Indonesia and in men in the Netherlands.

The prevalence of MetS among adults may vary for various reasons, such as altitude [37,38]; physical inactivity [38]; frequent intake of sugary foods; low educational level; overweight [39]; and even polypharmacy, especially in the elderly [40].

The high prevalence of MetS found in this study is closely related to the consumption pattern in Peru, in which there is a predominance of carbohydrates and a low presence of dietary fibre; the proportion of calories from fat and carbohydrates is also above the nutritional recommendations [41].

The adiposity indicators with the best AUC values in both genders observed in Table 3, such as VAI, WC, BRI, perimeter/height and relative fat mass, were the best predictors of MetS. This was observed in a similar way in a previous study carried out in Peru, in residents of the city of Lima and in the constitutional province of Callao, in which VAI, together with BRI, WC and BMI, presented the best AUC values in the prediction of MetS in both men and women [42]. In the present study, BMI was also an acceptable predictor; however, in the male gender, BMI did not reach an AUC higher than 0.8, compared to the other indicators considered as the best predictors.

VAI had the highest AUC compared to the other indicators of adiposity, and also been observed, most notably for WC, WHtR and BMI, in women with MetS and severe obesity and in patients with MetS and growth hormone deficiency [43,44]. This is because the inclusion of plasma triglycerides and HDL in the VAI formula, which are also indicators of MetS association, greatly improves the cardiometabolic risk prediction of central adiposity markers such as WC and other surrogates, such as BRI and WHtR, as well as BMI [45]. This aspect gives the VAI greater utility from a clinical and routine point of view; therefore, it should be implemented in hospital centres—not only in the city of Trujillo but throughout Peru. In addition to anthropometry, the evaluation of the lipid profile allows for better knowledge of its balance and, therefore, a greater preventive value against the risk of cardiovascular disease in the adult population, since VAI can distinguish a metabolically unhealthy obese person from a metabolically healthy obese person who has difficulty presenting an indicator of adiposity using purely anthropometric data such as WC, WHtR, BRI, percentage of relative fat mass and BMI.

The predictive value of the AUC found for VAI in the population of Trujillo is corroborated by a systematic review conducted by Bijari et al. [46] conducted in 2021, in which 21 out of 33 included studies reported the AUC value of VAI, with 76% indicating values above 0.8. The cut-off values for VAI in MetS differ in various locations and according to the diagnostic criteria used, ranging from 0.84 to 4.28 in men and from 1.15 to 4.11 in women. The cut-off value for VAI found in women for diagnosis of MetS in Trujillo is higher than that found by Gu et al. [47] in Shanghai, China (2.05), by Li et al. [48] in Americans (2.08), and by Mosad et al. [49] in Sudanese women (2.51). In men, the cut-off value is below those determined in studies in Venezuela [50] and in the USA [48], with values of 1.91 and 1.82, respectively, but higher than that determined in China (1.63) [47], with the application of ATPIII criteria in all these studies.

The higher AUC value for VAI in men compared to women was also observed in a study by Guo et al. [51], which suggests that these differences are related to the different regional distribution of adipose tissue according to sex, men have more visceral fat. Women gain some subcutaneous fat, then visceral fat increases at menopause, with a better prediction of MetS due to a higher Youden index and PPV, the latter suggesting that men are more sensitive to a positive diagnosis of MetS than women, as they have a higher cardiovascular risk level due to a greater accumulation of visceral fat in the abdominal region. Therefore, the analysis of the prediction of MetS using ROC curves for each adiposity indicator was not carried out with men and women in a single set, as this would bias the values, as would happen with the AUC values for relative fat mass and VAI, for example, which, unlike the other indicators, have formulae based on sex. For example, the cut-off points of the VAI for MetS are higher in women (2.57) than in men (1.73), an aspect that was also observed in the aforementioned studies [45,46,47] and that should, therefore, only be used as a reference for a given population, whether in a region or a country.

In women, the sensitivity for MetS is higher for BRI, WHtR, WC and relative fat mass percentage, as shown in Table 4, with values above 90% compared to men; this is also reflected in the slightly higher AUC values. These measures have also been shown to be predictive of visceral fat area in postmenopausal women [52]. This is consistent with the average age of the participants (47 years), as the decline in oestrogen during menopause leads to a redistribution of fat, which tends to accumulate in visceral adipose tissue [53]. Thus, in the case of women, VAI is very relevant from menopause onwards; therefore, it can be observed that it has a slightly higher AUC value but not as remarkable as BRI, WC, WHtR and the percentage of relative fat. To improve the predictive capacity of these indicators for MetS, it is important to consider additional information, together with other influential markers discussed below.

The best Youden index value observed for VAI in men in Trujillo’s population was also observed in a study by López-Gonzales et al. [54] in adults from nine communities in Spain and by Duan et al. [55] in Chinese adults, although with a value of 0.68 and good sensitivity and specificity values close to 80%. However, in Nigerians, Adejumo et al. [56] found a lower Youden index for VAI of 0.416, which was lower than other indicators, such as WC and BMI, with values of 0.591 and 0.549, respectively. This is probably due to a number of factors, including fitness genetics and ethnic differences.

Genetics significantly influences the distribution of body fat according to ethnicity. For example, plasma triglyceride concentrations are significantly lower in African Americans than in Caucasians. A lower level of triglycerides in the bloodstream suggests that more triglycerides have been absorbed into peripheral tissues. It is possible that people of Asian origin are at the lower end of the storage threshold for free fatty acids (FFAs) in their subcutaneous adipose tissue. As a result, FFAs continue to travel through the bloodstream to visceral adipose tissue (VAT) depots rather than being absorbed into subcutaneous tissues [53,57]. Japanese Americans have been found to have larger areas of visceral and hepatic adipose tissue compared to African Americans, and it has been suggested that polymorphisms in the PNPLA3 gene may be closely associated with hepatic steatosis [58]. These are probably the reasons why VAI levels were better associated with MetS in research from China and Spain and in the present study compared to Nigeria, so the genetic component is still a challenge to confirm.

Additionally, the Youden index for VAI was one of the best performers in predicting MetS in women, alongside other indicators, such as BRI, relative fat mass, waist-to-height ratio and WC. However, there have been studies where VAI has greater predictive power compared to the aforementioned indicators, as in a study by Chiu et al. [59] in Taiwanese adults.

Furthermore, it is worth noting that in the present study, VAI had the best PPV of around 64% compared to the other indicators, which were below 60%, and a very good NPV of close to 90%—surpassed in this case only by BRI, relative fat mass, WC and WHtR, as shown in Table 4. This indicates that VAI has a better balance in PPV and NPV values for MetS compared to these indicators and that it goes beyond the AUC value in its predictive ability for MetS.

There are other aspects to consider for possible biases in the PPV and NPV values, such as the presence of inflammatory markers involved in the metabolic syndrome. For example, adipocytes are cells with high metabolic activity that produce adipokines such as C-reactive protein, tumour necrosis factor alpha (TNFα) and interleukin-6 (IL-6), which increase the risk of diabetes and cardiovascular disease. Others, such as adiponectin, may theoretically reduce these risks [60].

Visceral adipose tissue, characterised by the presence of large adipocytes, is associated with poor metabolic health compared to subcutaneous adipose tissue; such adipocytes are more insulin resistant and have higher metabolic activity. These adipocytes have the ability to rapidly store fatty acids as triglycerides and release them as FFAs through lipolysis. Due to their resistance to insulin, this hormone cannot reduce the rate of lipolysis because it cannot inhibit triglyceride lipase and hormone-sensitive lipase [53].

It is proposed that FFAs generated by lipolysis of visceral adipose tissue enter the liver via the portal vein, increasing lipid synthesis, gluconeogenesis and insulin resistance. This can lead to hyperlipidaemia; glucose intolerance; hypertension; and, eventually, atherosclerosis [61]. Unhealthy lifestyles and insulin resistance in overweight individuals also increase hepatic FFAs levels and lipogenesis [62].

Triglyceride accumulation in hepatocytes leads to excessive release of inflammatory cytokines, which promotes hepatic steatosis and fibrosis. In addition, the production of very low-density lipoproteins (VLDLs) by triglyceride-laden hepatocytes stimulates Toll-like receptors and increases levels of hepatokines such as fetuin A and retinol transporter protein, all of which are involved in systemic inflammation [62].

In metabolism, VLDLs are transformed into LDLs by the action of lipoprotein lipase in the endothelial cells of blood vessels. Cholesterol ester transfer protein transfers triglycerides from VLDL to LDL, which allows hepatic lipases to hydrolyse these triglycerides and form smaller, denser LDL particles (sdLDLs) that can be oxidised in macrophages and, thus, contribute to atherosclerosis [63].

Decreased insulin sensitivity is not only associated with increased visceral fat but also with functional factors such as triglyceride and HDL levels. The condition of visceral obesity and dyslipidaemia of high triglycerides/low HDL cholesterol is explained by VAI [64], as it provides a better understanding of the modifications that develop in triglyceride-rich VLDLs to sdLDL formation and HDL depletion.

The study’s limitations include the fact that the participants belong to an urban district, so it would be necessary to consider rural populations to ensure that the VAI indicator can also be used for the Peruvian population in predicting MetS.

There is also likely to be a bias in the measurement of glycaemia by capillary due to hypotension, humidity and the hematocrit level [65], which is not expected at the time of sampling. However, it is known that the coefficient of variation in the determination of glycaemia starts from 120 mg/dL and is less than 4%, which is acceptable [66].

In the case of lipid profile measurement, the presence of hyperuricaemia and high concentrations of ascorbic acid, which can reduce the measurements, was not known. However, it is known that the coefficient of variation for the measurement of each parameter of the lipid profile and at the different concentration levels determined in venous blood samples is less than 4%, and this fact can also be established in the capillary sample [67]. Likewise, the biochemical measurements with capillaries were developed by the same evaluator with experience in laboratory analysis, an aspect that may reduce the bias of using a capillary sample.

Another limitation is the cross-sectional nature of the research, in which the concordance of the VAI value and the diagnosis of MetS could be strengthened in a longitudinal study with follow-up of the participants. This is because there are participants who, presenting two risk factors, may develop MetS in a short period of time. In this way, a longitudinal study could reduce false-negative and false-positive results and improve PPV and NPV, in addition to corroborating the cut-off value for MetS obtained in the present study.

Although other lifestyle factors were not considered in this study, a previous study found that most Trujillo residents did not consume alcohol (70%) or tobacco (90%) and that, in terms of diet, the majority consumed soft drinks (70%), about 56% consumed fruit and about 48% did not exercise. However, in a binary logistic regression model, these factors were not associated with metabolic syndrome, and only age and sex were risk factors [68]. However, it is important to mention that these lifestyles at another point in time may play a role in the development of metabolic syndrome, indicating the need for longitudinal studies.

It is also known that alcohol consumption, specifically beer consumption, aligns with the preferences of Latin Americans, and Peru was considered in the study by Brenes et al. [69]. High alcohol consumption is closely associated with an increase in visceral abdominal fat due to its high energy content (7.1 kcal/g) and because it can promote food consumption by inhibiting hormones related to satiety, such as leptin and glucagon-like peptide-1 [70].

This aspect contributes to the increase in VAI values in Peruvians and, therefore, a higher likelihood of presenting metabolic syndrome, especially in men compared to women. However, it is important to note that these lifestyle factors may play a role in the development of metabolic syndrome over time; therefore, longitudinal studies are needed.

Gender can be influenced by hormonal variations that can promote changes in PPV and NPV. In women, oestrogens inhibit the accumulation of visceral fat, which is more subcutaneous prior to menopause, leading to excess fat accumulation in the lower body (i.e., thighs and buttocks), which is reversed after menopause, leading to excess fat accumulation in the upper body, in which visceral fat plays a role, i.e., central obesity [71,72]. Similarly, menopause and the associated reduction in oestrogens lead to a significant increase in total cholesterol, triglycerides and LDL-C levels and a decrease in HDL-C levels compared with premenopausal women [73]. Therefore, these physiological and biochemical variations in PPV and NPV should be taken into account in VAI for their usefulness in predicting MetS.

The decrease in testosterone levels as men age causes a reduction in skeletal muscle mass and an increase in visceral fat, resulting in increased release of free fatty acids to the liver and leading to insulin resistance at the hepatic level. Therefore, it is important to evaluate these hormones to verify PPV and NPV results when using VAI [74].

Regarding the specific use of the adiposity indicators used in the present study, other indicators, such as the abdominal volume index (AVI) and body adiposity index (BAI), were not taken into account because hip circumference was not determined, nor the hepatic steatosis index, since aspartate aminotransferase (AST) and alanine aminotransferase (ALT) data were not available for calculation [72].

As a strength, the study’s result is finding an indicator with greater discriminative capacity for the presence of MetS diagnosed by harmonised MetS criteria that is even suitable for the Latin American population by using the abdominal circumference cut-off established by ALAD.

## 5. Conclusions

It is concluded that VAI is the adiposity indicator that best predicts MetS in the inhabitants of the city of Trujillo (both men and women), as it has better values of AUC, PPV, NPV and the Youden Index compared to other adiposity indicators; therefore, it can be used for prevention and diagnosis of MetS. VAI is a good quantitative and qualitative indicator to consider due to its adequate interpretation of the balance in lipid metabolism and the activity of visceral adipose tissue, factors involved in MetS and, therefore, cardiovascular risk.

## Figures and Tables

**Table 1:** Baseline characteristics of male participants according to presence of MetS.

Baseline Characteristics	Without MetS		With MetS		Significance (*p*)
	Median ± IQR	X ± SD	Median ± IQR	X ± SD	
Waist circumference (cm)	89.90 ± 9.70	90.76 ± 8.92	100 ± 7.70	101.89 ± 10.75	0.00
Glycaemia (mg/dL)	97 ± 8	97.83 ± 5.39	104 ± 7.50	106.36 ± 19.38	0.00
Systolic blood pressure (mmHg)	112 ± 11	111.66 ± 11.45	121 ± 20	120.88 ± 15.38	0.00
Diastolic blood pressure (mmHg)	73 ± 8	71.8 ± 7.48	78 ± 14.50	77.67 ± 10.63	0.00
Cholesterol (mg/dL)	172 ± 56.50	175.54 ± 35.51	190 ± 55	190.59 ± 38.19	0.04
Triglycerides (mg/dL)	98 ± 33	100.95 ± 35.78	168 ± 109	197.72 ± 111.78	0.00
HDL (mg/dL)	36 ± 14	39.29 ± 13.02	30 ± 13	29.5 ± 8.27	0.00
LDL (mg/dL)	118 ± 45.50	116.54 + 30.65	120 ± 46.50	135.90 ± 103.89	0.23
Risk factors	1 ± 1	1.32 ± 0.69	3 ± 1	3.52 ± 0.68	0.00
Weight (kg)	73.50 ± 14.15	74.44 ± 11.21	82.90 ± 13	85.95 ± 16.16	0.00
Tricipital skinfold (mm)	12.20 ± 6.95	13.32 ± 5.10	16 ± 7.40	16.96 ± 7.42	0.00
Bicipital skinfold (mm)	6.5 ± 4.60	6.98 ± 4	7.20 ± 6.60	8.65 ± 5.46	0.14
Suprailiac skinfold (mm)	15.50 ± 10.05	17.23 ± 7.62	24 ± 10	23.94 ± 7.91	0.00
Subscapular skinfold (mm)	19.80 ± 9.45	20.23 ± 8.09	24 ± 9.50	25.31 ± 7.60	0.00
Height (m)	1.67 ± 0.09	1.66 ± 0.07	1.67 ± 0.09	1.67 ± 0.07	0.93

IQR: Interquartile range (Mann–Whitney U test was used).

**Table 2:** Baseline characteristics of female participants according to presence of MetS.

Baseline Characteristics	Without MetS		With MetS		Significance (*p*)
	Median IQR	X ± SD	Median IQR	X ± SD	
Waist circumference (cm)	83 ± 9.88	83.83 ± 9.37	95 ± 11.10	95.44 ± 7.67	0.00
Glycaemia (mg/dL)	97 ± 9	97.05 ± 7.54	104 ± 11	113.28 ± 34.17	0.00
Systolic blood pressure (mmHg)	105 ± 20	107.49 ± 15.21	119 ± 25	120.23 ± 18.21	0.00
Diastolic blood pressure (mmHg)	70 ± 12	70.46 ± 10.15	79 ± 15	79.04 ± 10.52	0.00
Cholesterol (mg/dL)	200 ± 61	205.10 ± 49.41	219 ± 82	225.96 ± 54.09	0.02
Triglycerides (mg/dL)	104 ± 49	114.51 ± 57.64	176 ± 150	205.83 ± 108.8	0.00
HDL (mg/dL)	48.50 ± 20	50.44 ± 15.13	37 ± 10	37.55 ± 8.14	0.00
LDL (mg/dL)	130.5 ± 56.25	133.33 ± 40.82	144 ± 69	149.78 ± 49.36	0.07
Risk factors	1 ± 1	1.30 ± 0.74	4 ± 1	3.68 ± 8.42	0.00
Weight (kg)	61.50 ± 10.2	61.32 ± 8.42	72.10 ± 13.7	72.7 ± 10.6	0.00
Tricipital skinfold (mm)	20 ± 7.23	20.7 ± 5.33	23.6 ± 7	23.8 ± 6.37	0.00
Bicipital skinfold (mm)	11 ± 7.43	11.42 ± 6.35	14 ± 7	15.16 ± 6.15	0.00
Suprailiac skinfold (mm)	21.75 ± 9.40	21.89 ± 6.42	28 ± 9.10	28.11 ± 7.07	0.00
Subscapular skinfold (mm)	22.45 ± 8.70	23.17 ± 7.94	29 ± 11	29.55 ± 7.58	0.00
Height (m)	1.54 ± 0.08	1.54 ± 006	1.55 ± 0.06	1.54 ± 0.05	0.86

IQR: Interquartile range (Mann–Whitney U test was used).

**Table 3:** Prevalence of MetS according to age and gender in citizens of Trujillo (2023).

Factors		MetS				Total		Significance (*p*)
		No	%	Yes	%		%	
Age	Under 50	92	34.5%	57	21.3%	149	55.8%	0.06
	50 and older	59	22.1%	59	22.1%	118	44.2%	
Gender	Female	110	41.2%	47	17.6%	157	58.8%	0.00
	Male	41	15.4%	69	25.8%	110	41.2%	
Total		151	56.6%	116	43.4%	267	100.0%	

**Table 4:** Area under the curve and cut-off points for adiposity indicators in the prediction of MetS.

Indicators of Adiposity	Area Under the Curve	Sig.	95% Confidence Interval		Cut-Off Point	Sens	Specif	Youden Index	PPV	NPV
			Lower Limit	Upper Limit						
Men										
BMI	0.769	0.000	0.674	0.864	27.86	82.61	68.29	0.51	81.43	70
ABSI	0.570	0.222	0.458	0.68	0.06	55.07	63.41	0.18	71.7	45.61
BRI	0.81	0.000	0.721	0.9	4.38	89.86	63.41	0.53	80.52	78.79
VAI	0.875	0.000	0.809	0.941	1.73	91.3	73.17	0.64	85.14	83.33
CI	0.743	0.000	0.648	0.838	1.29	62.32	75.61	0.38	81.13	54.39
%Fat	0.721	0.000	0.62	0.822	28.96	57.97	80.49	0.38	83.33	53.23
RFM	0.81	0.000	0.721	0.899	29.15	78.26	75.61	0.54	84.38	67.39
WC	0.824	0.000	0.736	0.912	95.9	78.26	80.49	0.59	87.1	68.75
WHtR	0.803	0.000	0.714	0.893	0.60	55.07	87.8	0.43	88.37	53.73
Women										
BMI	0.818	0.000	0.747	0.889	27.15	89.36	67.27	0.57	53.85	93.67
ABSI	0.607	0.035	0.513	0.701	0.08	34.04	76.36	0.1	38.1	73.04
BRI	0.832	0.000	0.767	0.898	4.59	93.62	68.18	0.62	55.7	96.15
VAI	0.858	0.000	0.797	0.919	2.57	78.72	80.91	0.6	63.79	89.9
CI	0.742	0.000	0.664	0.819	1.25	63.83	69.09	0.33	46.88	81.72
%Fat	0.796	0.000	0.723	0.870	37.44	78.72	69.09	0.48	52.11	88.37
RFM	0.832	0.000	0.766	0.897	40.5	91.49	70	0.61	56.58	95.06
WC	0.856	0.000	0.794	0.918	87.75	93.62	71.82	0.65	58.67	96.34
WHtR	0.831	0.000	0.766	0.897	0.56	93.62	68.18	0.62	55.7	96.15

Sig. = significance; Sens = sensitivity; Specif = specificity.

## Data Availability

The data presented in this study are available from the corresponding author upon request due to ethical reasons.

## Supplementary Materials

The following supporting information can be downloaded at: https://www.mdpi.com/article/10.3390/medicina61030419/s1, File S1. Data collection form.

## Abbreviations

The following abbreviations are used in this manuscript:

ABSI	Body shape index
ALAD	Latin American Diabetes Association
ATPIII	Adult Treatment Panel III
AUC	Area under the curve
BMI	Body mass index
BRI	Body roundness index
CI	Conicity index
FFAs	Free fatty acids
HDL	High-density lipoprotein
LDL	Low-density lipoprotein
MetS	Metabolic syndrome
NPV	Negative predictive value
PPV	Positive predictive value
RFM	Relative fat mass
ROC	Receiver operating characteristic curve
TG	Triglyceride
VAI	Visceral adiposity index
VAT	Visceral adipose tissue
WC	Waist circumference
WHtR	Waist-to-height ratio

## References

[1] Raimi T.H., Dele-Ojo B.F., Dada S.A., Fadare J.O., Ajayi D.D., Ajayi E.A., Ajayi O.A. (2021). Triglyceride-Glucose Index and Related Parameters Predicted Metabolic Syndrome in Nigerians. Metab. Syndr. Relat. Disord..

[2] Pan W.H., Yeh W.T., Weng L.C. (2008). Epidemiology of Metabolic Syndrome in Asia. Asia Pac. J. Clin. Nutr..

[3] Arsentales V., Tenorio M., Bernabé A. (2019). Association Between Work-Related Physical Activity and Metabolic Syndrome: A Population-Based Study in Peru. Rev. Chil. Nutr..

[4] Tejada Lopez Y.O., Choquehuanca Zambrano G.M., Goicochea Ríos E.D.S., Vicuña Villacorta J.E., Olga Yanet G.A. (2020). Perfil Clínico-Epidemiológico del Síndrome Metabólico en Adultos Atendidos en el Hospital I Florencia de Mora EsSALUD. Horiz. Médico.

[5] Espinoza-Rivera S., Andrea Rivera P., Ballinas Sueldo Y. (2023). Prevalencia y Componentes del Síndrome Metabólico Premórbido en Trabajadores Asegurados al Seguro Social de Salud en una Zona de Altitud Moderada en Perú. Acta Med. Peru..

[6] Noubiap J., Nansseu J., Lontchi-Yimagou E., Nkeck J., Nyaga U., Ngouo A., Tounouga D.N., Tianyi F.-L., Foka A.J., Ndoadoumgue A.L. (2022). Geographic Distribution of Metabolic Syndrome and Its Components in the General Adult Population: A Meta-Analysis of Global Data from 28 Million Individuals. Diabetes Res. Clin. Pract..

[7] Mittal B. (2019). Subcutaneous adipose tissue & visceral adipose tissue. Indian J. Med. Res..

[8] Bryce-Moncloa A., Alegría-Valdivia E., Martin-San Martin S., Mauricio G. (2017). Obesidad y riesgo de enfermedad cardiovascular. An. Fac. Med..

[9] Almeida E.D.P., Sabino Pinho C.P., Leão A.P.D., Rodrigues I.G., Diniz A.D.S., de Arruda I.K.G. (2018). Razón entre grasa visceral y subcutánea como predictor de alteraciones cardiometabólicas. Rev. Chil. Nutr..

[10] Shuster A., Patlas M., Pinthus J.H., Mourtzakis M. (2012). The Clinical Importance of Visceral Adiposity: A Critical Review of Methods for Visceral Adipose Tissue Analysis. Br. J. Radiol..

[11] Després J.P. (2012). Body Fat Distribution and Risk of Cardiovascular Disease: An Update. Circulation.

[12] Grundy S.M., Neeland I.J., Turer A.T., Vega G.L. (2013). Waist Circumference as Measure of Abdominal Fat Compartments. J. Obes..

[13] Woolcott O.O., Bergman R.N. (2019). Relative Fat Mass as an Estimator of Whole-Body Fat Percentage Among Children and Adolescents: A Cross-Sectional Study Using NHANES. Sci. Rep..

[14] Kobo O., Leiba R., Avizohar O., Karban A. (2019). Relative Fat Mass Is a Better Predictor of Dyslipidemia and Metabolic Syndrome than Body Mass Index. Cardiovasc. Endocrinol. Metab..

[15] Ahmad M.N., Haddad F.H. (2015). Suitability of Visceral Adiposity Index as a Marker for Cardiometabolic Risks in Jordanian Adults. Nutr. Hosp..

[16] Baveicy K., Mostafaei S., Darbandi M., Hamzeh B., Najafi F., Pasdar Y. (2020). Predicting Metabolic Syndrome by Visceral Adiposity Index, Body Roundness Index and a Body Shape Index in Adults: A Cross-Sectional Study from the Iranian Rancd Cohort Data. Diabetes Metab. Syndr. Obes..

[17] Sousa N.C., Marques F.R.D.M., Pires G.A.R., Scardoelli M.G.D.C., Rêgo A.D.S., Radovanovic C.A.T., Salci M.A. (2020). Conicity Index in People with Hypertension Followed in the Brazil’s Family Health Strategy. Rev. Bras. Enferm..

[18] Raya-Cano E., Molina-Recio G., Romero-Saldaña M., Álvarez-Fernández C., Hernández-Reyes A., Molina-Luque R. (2020). Comparación de Índices Antropométricos, Clásicos y Nuevos, para el Cribado de Síndrome Metabólico en Población Adulta Laboral. Rev. Esp. Salud Publica.

[19] Xu J., Zhang L., Wu Q., Zhou Y., Jin Z., Li Z., Zhu Y. (2021). Body Roundness Index Is a Superior Indicator to Associate with the Cardio-Metabolic Risk: Evidence from a Cross-Sectional Study with 17,000 Eastern-China Adults. BMC Cardiovasc. Disord..

[20] Sánchez-Rodríguez M.A. (2022). ¿Cómo Puedo Calcular el Tamaño de la Muestra? Importancia en la Calidad y la Validez en la Investigación en Ciencias de la Salud. Casos Revis. Salud.

[21] Ministerio de Salud—Perú (2013). Guía Técnica para la Valoración Nutricional Antropométrica de la Persona Adulta Mayor.

[22] Ministerio de Salud—Perú (2012). Guía Técnica para la Valoración Nutricional Antropométrica de la Persona Adulta.

[23] López-García R., Lagunes-Carrasco J., Banda-Sauceda N., Durazo-Terán L. (2018). Comparación de la grasa corporal a través de dos métodos de medición en futbolistas. Rev. Técnicas Enferm..

[24] Sprecher D.L., Frolkis J.P. (2001). Using the New Cholesterol Guidelines in Everyday Practice. Cleve. Clin. J. Med..

[25] Lizarzaburu J. (2014). Síndrome Metabólico: Concepto y Aplicación Práctica. An. Fac. Med..

[26] Gotthelf S., Rivas P. (2018). Síndrome Metabólico y Obesidad Según Criterios IDF/ALAD en Adultos de la Ciudad de Salta. Rev. Salud Publica.

[27] IBM Corp (2019). IBM SPSS Statistics for Windows.

[28] Castro M. (2019). Biostatistics Applied in Clinical Research: Basic Concepts. Rev. Med. Clin. Condes.

[29] Cerda J., Cifuentes L. (2012). Using ROC Curves in Clinical Investigation. Theoretical and Practical Issues. Rev. Chil. Infectol..

[30] Asociación Médica Mundial Declaración de Helsinki de la AMM-Principios Éticos para las Investigaciones Médicas en Seres Humanos. https://www.wma.net/es/policies-post/declaracion-de-helsinki-de-la-amm-principios-eticos-para-las-investigaciones-medicas-en-seres-humanos.

[31] Ramezankhani A., Azizi F., Hadaegh F. (2022). Gender Differences in Changes in Metabolic Syndrome Status and Its Components and Risk of Cardiovascular Disease: A Longitudinal Cohort Study. Cardiovasc. Diabetol..

[32] Zou Y., Kuang M., Zhong Y., Jiang C. (2023). Remnant Cholesterol Can Identify Individuals at Higher Risk of Metabolic Syndrome in the General Population. Sci. Rep..

[33] Datta Banik S.D., Pacheco-Pantoja E., Lugo R., Gómez-De-Regil L., Aké R.C., González R.M.M., Solis A.L.G. (2021). Evaluation of Anthropometric Indices and Lipid Parameters to Predict Metabolic Syndrome Among Adults in Mexico. Diabetes Metab. Syndr. Obes..

[34] Li N., Fu J., Koonen D.P., Kuivenhoven J.A., Snieder H., Hofker M.H. (2014). Are Hypertriglyceridemia and Low HDL Causal Factors in the Development of Insulin Resistance?. Atherosclerosis.

[35] Mazloomzadeh S., Rashidi Khazaghi Z., Mousavinasab N. (2018). The Prevalence of Metabolic Syndrome in Iran: A Systematic Review and Meta-Analysis. Iran J. Public Health.

[36] Sigit F.S., Tahapary D.L., Trompet S., Sartono E., Willems Van Dijk K., Rosendaal F.R., de Mutsert R. (2020). The Prevalence of Metabolic Syndrome and Its Association with Body Fat Distribution in Middle-Aged Individuals from Indonesia and the Netherlands: A Cross-Sectional Analysis of Two Population-Based Studies. Diabetol. Metab. Syndr..

[37] Sanchez-Samaniego G., Mäusezahl D., Carcamo C., Probst-Hensch N., Verastegui H., Hartinger S.M. (2022). Metabolic Syndrome in Rural Peruvian Adults Living at High Altitudes Using Different Cookstoves. PLoS ONE.

[38] Juna C.F., Cho Y.H., Joung H. (2020). Low Elevation and Physical Inactivity Are Associated with a Higher Prevalence of Metabolic Syndrome in Ecuadorian Adults: A National Cross-Sectional Study. Diabetes Metab. Syndr. Obes..

[39] Zoraski H., Fiametti M., Santos R.D., Gregoletto M.L.O., Cremonese C. (2017). Metabolic Syndrome in Elderly from Nova Roma do Sul, RS: Prevalence and Associated Factors. ABCS Health Sci..

[40] Tavares D.S., Gomes N.C., Rodriguês L.R., Tavares D.M. (2018). Profile of Elderly Persons with Metabolic Syndrome and Factors Associated with Possible Drug Interactions. Rev. Bras. Geriatr. Gerontol..

[41] Adams Ubaldo K.J., Chirinos J.L. (2018). Prevalence of Risk Factors for Metabolic Syndrome and Its Components in Community Kitchen Users in a District in Lima, Peru. Rev. Peru Med. Exp. Salud Publica.

[42] Stefanescu A., Revilla L., Lopez T., Sanchez S.E., Williams M.A., Gelaye B. (2020). Using A Body Shape Index (ABSI) and Body Roundness Index (BRI) to Predict Risk of Metabolic Syndrome in Peruvian Adults. J. Int. Med. Res..

[43] Lazzer S., D’Alleva M., Isola M., De Martino M., Caroli D., Bondesan A., Marra A., Sartorio A. (2023). Cardiometabolic Index (CMI) and Visceral Adiposity Index (VAI) Highlight a Higher Risk of Metabolic Syndrome in Women with Severe Obesity. J. Clin. Med..

[44] Qing L., Wei R., Chan L., Xiaoya Z., Xin X. (2017). Sensitivity of Various Body Indices and Visceral Adiposity Index in Predicting Metabolic Syndrome Among Chinese Patients with Adult Growth Hormone Deficiency. J. Endocrinol. Investig..

[45] Barazzoni R., Gortan Cappellari G., Semolic A., Ius M., Zanetti M., Gabrielli A., Vinci P., Guarnieri G., Simon G. (2019). Central Adiposity Markers, Plasma Lipid Profile and Cardiometabolic Risk Prediction in Overweight-Obese Individuals. Clin. Nutr..

[46] Bijari M., Jangjoo S., Emami N., Raji S., Mottaghi M., Moallem R., Jangjoo A., Saberi A. (2021). The Accuracy of Visceral Adiposity Index for the Screening of Metabolic Syndrome: A Systematic Review and Meta-Analysis. Int. J. Endocrinol..

[47] Gu Z., Zhu P., Wang Q., He H., Xu J., Zhang L., Li D., Wang J., Hu X., Ji G. (2018). Obesity and Lipid-Related Parameters for Predicting Metabolic Syndrome in Chinese Elderly Population. Lipids Health Dis..

[48] Li Y., Zheng R., Li S., Cai R., Ni F., Zheng H., Hu R., Sun T. (2022). Association Between Four Anthropometric Indexes and Metabolic Syndrome in US Adults. Front. Endocrinol..

[49] Mosad A., Elfadil G., Elhassan S., Elbashir Z., Husain N., Karar T., Elfaki E. (2023). Diagnostic Performance Using Obesity and Lipid-Related Indices and Atherogenic Index of Plasma to Predict Metabolic Syndrome in the Adult Sudanese Population. Niger. J. Clin. Pract..

[50] Bermúdez V.J., Salazar J., Añez R., Rivas-Ríos J.R., Chávez-Castillo M., Torres W., Núñez V., Mejías J., Wilches-Durán S., Cerda M. (2017). Optimal Cutoff for Visceral Adiposity Index in a Venezuelan Population: Results from the Maracaibo City Metabolic Syndrome Prevalence Study. Rev. Argent. Endocrinol. Metab..

[51] Guo S.X., Zhang X.H., Zhang J.Y., He J., Yan Y.Z., Ma J.L., Ma R.L., Guo H., Mu L.T., Li S.G. (2016). Visceral Adiposity and Anthropometric Indicators as Screening Tools of Metabolic Syndrome Among Low Income Rural Adults in Xinjiang. Sci. Rep..

[52] Gondim Pitanga F.J., Seara Pitanga C.P., Calçada Dias Gabriel R.E., Cristina Beck C., Rodrigues Moreira M.H. (2015). Anthropometry to Identify High Visceral Fat Area in Postmenopausal Women. Nutr. Hosp..

[53] Dhokte S., Czaja K. (2024). Visceral Adipose Tissue: The Hidden Culprit for Type 2 Diabetes. Nutrients.

[54] López-González A.A., Jover A.M., Martínez C.S., Artal P.M., Bote S.A., Jané B.A., Ramírez-Manent J.I. (2022). The CUN-BAE, Deurenberg Fat Mass, and Visceral Adiposity Index as Confident Anthropometric Indices for Early Detection of Metabolic Syndrome Components in Adults. Sci. Rep..

[55] Duan Y., Zhang W., Li Z., Niu Y., Chen Y., Liu X., Dong Z., Zheng Y., Chen X., Feng Z. (2022). Predictive Ability of Obesity- and Lipid-Related Indicators for Metabolic Syndrome in Relatively Healthy Chinese Adults. Front. Endocrinol..

[56] Adejumo E.N., Adejumo A.O., Azenabor A., Ekun A.O., Enitan S.S., Adebola O.K., Ogundahunsi O.A. (2019). Anthropometric Parameter That Best Predict Metabolic Syndrome in South West Nigeria. Diabetes Metab. Syndr. Clin. Res. Rev..

[57] Steiner B.M., Berry D.C. (2022). The Regulation of Adipose Tissue Health by Estrogens. Front. Endocrinol..

[58] Lim U., Monroe K.R., Buchthal S., Fan B., Cheng I., Kristal B.S., Lampe J.W., Hullar M.A., Franke A.A., Stram D.O. (2019). Propensity for Intra-abdominal and Hepatic Adiposity Varies Among Ethnic Groups. Gastroenterology.

[59] Chiu T.H., Huang Y.C., Chiu H., Wu P.Y., Chiou H.Y.C., Huang J.C., Chen S.-C. (2020). Comparison of Various Obesity-Related Indices for Identification of Metabolic Syndrome: A Population-Based Study from Taiwan Biobank. Diagnostics.

[60] Zaha D., Vesa C., Uivarosan D., Bratu O., Fratila O., Tit D., Pantis C., Diaconu C.C., Bungau S. (2020). Influence of Inflammation and Adipocyte Biochemical Markers on the Components of Metabolic Syndrome. Exp. Ther. Med..

[61] Patel P., Abate N. (2013). Body Fat Distribution and Insulin Resistance. Nutrients.

[62] Jichitu A., Bungau S., Stanescu A.M.A., Vesa C.M., Toma M.M., Bustea C., Iurciuc S., Rus M., Bacalbasa N., Diaconu C.C. (2021). Non-Alcoholic Fatty Liver Disease and Cardiovascular Comorbidities: Pathophysiological Links, Diagnosis, and Therapeutic Management. Diagnostics.

[63] Wang X., Wang L., Cao R., Yang X., Xiao W., Zhang Y., Ye P. (2021). Correlation between Small and Dense Low-Density Lipoprotein Cholesterol and Cardiovascular Events in Beijing Community Population. J. Clin. Hypertens..

[64] Amato M.C., Giordano C., Galia M., Criscimanna A., Vitabile S., Midiri M., Galluzzo A., AlkaMeSy Study Group (2010). Visceral Adiposity Index: A Reliable Indicator of Visceral Fat Function Associated with Cardiometabolic Risk. Diabetes Care.

[65] Gygliola P., Tarquino G., Chambi E., Averanga K., Salcedo L. (2020). Glucose Determination: The Use of Glucometers as a Rapid Test of Analysis. J. Selva Andina Res. Soc..

[66] Roche Diabetes Care (2019). Accu Chek Performa Nano Tests.

[67] ACON Laboratories, Inc. (2013). Dispositivos para Examen de Colesterol.

[68] Díaz H., Yupari I. (2022). Modelo de Predicción para Síndrome Metabólico en Adultos de Trujillo, Perú. Rev. Haban. Cienc. Médicas.

[69] Brenes J.C., Gómez G., Quesada D., Kovalskys I., Rigotti A., Cortés L., García M.C.Y., Liria-Domínguez R., Herrera-Cuenca M., Guajardo V. (2021). Contribución del alcohol a la ingesta total de energía y su asociación con el estado nutricional y la calidad de la dieta en ocho países latinoamericanos. Int. J. Medio Ambiente. Res. Salud Pública.

[70] Lampenius I., Harjutsalo V., Parente E.B., Groop P.H., Grupo de estudio FinnDiane (2023). Asociaciones entre el consumo de alcohol y la distribución de la grasa corporal en la diabetes tipo 1. Diabetes Res. Clin. Pract..

[71] Quaye L., Owiredu W.K.B.A., Amidu N., Dapare P.P.M., Adams Y. (2019). Comparative Abilities of Body Mass Index, Waist Circumference, Abdominal Volume Index, Body Adiposity Index, and Conicity Index as Predictive Screening Tools for Metabolic Syndrome among Apparently Healthy Ghanaian Adults. J. Obes..

[72] Lin I.-T., Lee M.-Y., Wang C.-W., Wu D.-W., Chen S.-C. (2021). Gender Differences in the Relationships among Metabolic Syndrome and Various Obesity-Related Indices with Nonalcoholic Fatty Liver Disease in a Taiwanese Population. Int. J. Environ. Res. Public Health.

[73] Hamza A., Elfaki E., Abbas M., Ibrahim M., Mohamed E., Karar T. (2019). Assessment of Plasma Lipid Profile Among Sudanese Menopausal Women in Khartoum State-Sudan. Biomed. Pharmacol. J..

[74] Chung T.H., Kwon Y.J., Lee Y.J. (2020). High Triglyceride to HDL Cholesterol Ratio Is Associated with Low Testosterone and Sex Hormone-Binding Globulin Levels in Middle-Aged and Elderly Men. Aging Male.

