# Supplementary material for: Predictive Capacity of Different Indicators of Adiposity for Metabolic Syndrome in Adults in the City of Trujillo, Peru
Source: Medicina (Kaunas). 2025 Feb 27;61(3):419. doi: 10.3390/medicina61030419 (PMC11943740; doi:10.3390/medicina61030419)
Supplement: Supplementary file 1 [file medicina-61-00419-s001.zip › medicina-3464110-supplementary.pdf]

Data collection form

Physiological and biochemical characteristics

Last Name and First Name: …………………………………………………………….

Age: ………………….

Weight: ………………….Kg

Height: …………………… m

BMI: …………………….Kg/m^2

Abdominal circumference: .... cm

Blood pressure (BP):

Systolic BP: .... mmHg   Diastolic BP: .... mg/dL

Glycaemia: .... mg/dL

Lipid Profile

Total Cholesterol: .... mg/dL   Triglycerides: ....mg/dL

HDL: ....mg/dL   LDL: .... mg/dL

Number of risk factors: 0 (.......) 1 (......) 2(......) ≥3 (......)

Anthropometric data:

Weight: ………….. kg

Height: ………….. m

BMI: …………… kg/m²

Waist circumference: ………… cm

Tricipital fold: ……………. mm

Bicipital fold: …………………… mm

Suprailiac fold: ……………… mm

Subscapular fold: ……………… mm

Adiposity indicators:

Body fat percentage: ………………

WC/Height (WHtR) : ………….

BRI: ………………………………

ABSI: ……………………………

VAI: …………………………….

CI: ………………………………

RFM: ……………………………
